# Supplementary material for: Increasing the Activity of the High-Fidelity SpyCas9 Form in Yeast by Directed Mutagenesis of the PAM-Interacting Domain
Source: Int J Mol Sci. 2023 Dec 28;25(1):444. doi: 10.3390/ijms25010444 (PMC10779060; doi:10.3390/ijms25010444)
Supplement: Supplementary file 1 [file ijms-25-00444-s001.zip › ijms-2779289-supplementary.pdf]

Table S1. Oligonucleotides used in this work

Name	Sequence (5'→3')
p414-Cas9-48-XhoI-F	AAGCGTCAAGGAACTGCTGGG
p414-Cas9-55-EcoRI-F	CCGAAGATAATGAGCAGAAGCA
E1341H+A1345L-F	GAAAGCGGTACACCTCTACAAAACACGTCCTGGACCTCACACTGATTC
E1341H+A1345L-R	ATTGACTGATGAATCAGTGTGAGGTCCAGGACGTGTTTTGTAGAGGTGTACC
E1341D-F	GAAAGCGGTACACCTCTACAAAAGATGTCCTG
E1341D-R	GTGGCGTCCAGGACATCTTTTGTAGAGGTGTACC
E1341H-F	GAAAGCGGTACACCTCTACAAAACACGTCCTG
E1341H-R	GTGGCGTCCAGGACGTGTTTTGTAGAGGTGTACC
A1345L-F	CTCTACAAAAGAGGTCCTGGATCTCACACTGATTC
A1345L-R	ATTGACTGATGAATCAGTGTGAGATCCAGG
A1345P-F	CTCTACAAAAGAGGTCCTGGATCCCACACTGATTC
A1345P-R	ATTGACTGATGAATCAGTGTGGGATCCAGG
